# Association between elevated serum uric acid levels and high estimated glomerular filtration rate with reduced risk of low muscle strength in older people: a retrospective cohort study

**DOI:** 10.1186/s12877-023-04374-3

**Published:** 2023-10-11

**Authors:** Yu Cheng Huang, Si Liang Chen, Ying Dong, Ying Shi

**Affiliations:** 1 Shi’s Center of Orthopedics and Traumatology, Shuguang Hospital, Shanghai University of Traditional Chinese Medicine, Shanghai, China; 2 Institute of Traumatology & Orthopedics, Shanghai Academy of Traditional Chinese Medicine, Shanghai, 201220 China; 3 School of Public Health, Shanghai University of Traditional Chinese Medicine, Shanghai, China

**Keywords:** Uric acid, Muscle strength, Sarcopenia, eGFR

## Abstract

**Background:**

We aimed to investigate the interaction between serum uric acid (SUA) levels with estimated glomerular filtration rate (eGFR) to low muscle strength (LMS) among older people in China.

**Methods:**

Cohort data were obtained from China Health and Retirement Longitudinal Study (CHARLS) in 2011 and 2015. A total of 2,822 community-dwelling adults aged 60 and above were enrolled for the follow-up. Serum uric acid was collected after 8 h of fasting, and handgrip strength was measured with a dynamometer. eGFR was calculated with an equation based on the Chinese population. A generalized additive model was employed for interaction analysis and progressively adjusted confounders.

**Results:**

During the follow-up, a total of 659 individuals were excluded due to the lack of grip strength data, leaving 2,163 participants for analysis. Despite the protective effect of high uric acid against low muscle strength, especially in older females, it is not statistically significant (OR = 0.69, 95%CI = 0.45–1.04, P = 0.075). Following the progressive adjustment of covariates, the association between higher eGFR and elevated SUA levels remained statistically significant in females, showing a reduced odds ratio with low muscle strength (OR = 0.82, 95%CI = 0.70–0.97, P = 0.021). However, this trend was not observed in male participants.

**Conclusions:**

This Chinese population-based cohort study suggests that among older females, a higher serum uric acid level combined with a higher estimated glomerular filtration rate is linked to a reduced risk of low muscle strength. This implies that the relationship between high serum uric acid levels and the risk of low grip strength might differ by gender.

**Supplementary Information:**

The online version contains supplementary material available at 10.1186/s12877-023-04374-3.

## Introduction

With advancing age, the human body is accompanied by a series of physiological changes, including the loss of skeletal muscle mass and strength, which is defined as sarcopenia [1]. Muscle strength, especially handgrip strength, is a crucial parameter for assessing and diagnosing sarcopenia [2]. The declined strength as a predicted factor is also associated with an increased risk of falls [3], fracture [4], cancer [5], and even mortality [6, 7]. Despite many factors affecting the loss of muscle strength in older people, the accumulation of reactive oxygen species (ROS) is one of the reasons for the age-related functional losses [8], causing oxidative protein damage and diminishing muscular strength [9].

Serum uric acid (SUA) is an end-product of purine metabolism and is believed to possess both pro-oxidant and antioxidant properties [10]. Furthermore, SUA has been proposed as a dependable indicator of oxidative stress [11]. However, the existing body of research on the association between SUA levels and low muscle strength (LMS) has produced inconsistent outcomes. Some studies have suggested the existence of an optimal range of SUA levels that correlates with improved grip strength [12, 13]. In contrast, a cross-sectional study utilizing data from the WCHAT (West China Health and Aging Trend) suggested a positive association between SUA levels and muscle strength [14]. Similarly, a NHANES (National Health and Nutrition Examination Survey) study also supported the notion that higher SUA levels could serve as a protective factor for muscle strength in older people [15].

Conversely, findings from the PRO.V.A. (*Progetto Veneto Anziani*) cohort study demonstrated that hyperuricemia was associated with reduced physical performance in older people, particularly concerning handgrip strength among men [16]. These conflicting results underscore the need for further comprehensive investigation and analysis to elucidate the potential role of SUA in contributing to age-related decline in muscle strength. As such, it remains unclear whether the causal relationship between high uric acid levels and increased grip strength, especially since most existing literature is based on cross-sectional studies that cannot determine causality.

Moreover, the relationship between SUA and muscle strength is intricately linked to kidney function. Approximately 70% of uric acid excretion is regulated through renal mechanisms. Thus, considering the different stages of kidney function related to SUA levels is crucial when studying the association with muscle strength. It has been observed that muscle strength tends to decrease significantly in individuals with lower estimated glomerular filtration rate (eGFR) [17], suggesting a potential interplay between SUA levels and kidney function in their influence on muscle strength.

Therefore, this study aimed to use data from the CHARLS (China Health and Retirement Longitudinal Study) to investigate the relationship between UA and low muscle strength and to explore the relationship between high eGFR level with SUA levels to low muscle strength in older people in China.

## Methods

### Study population

**Fig. 1:**
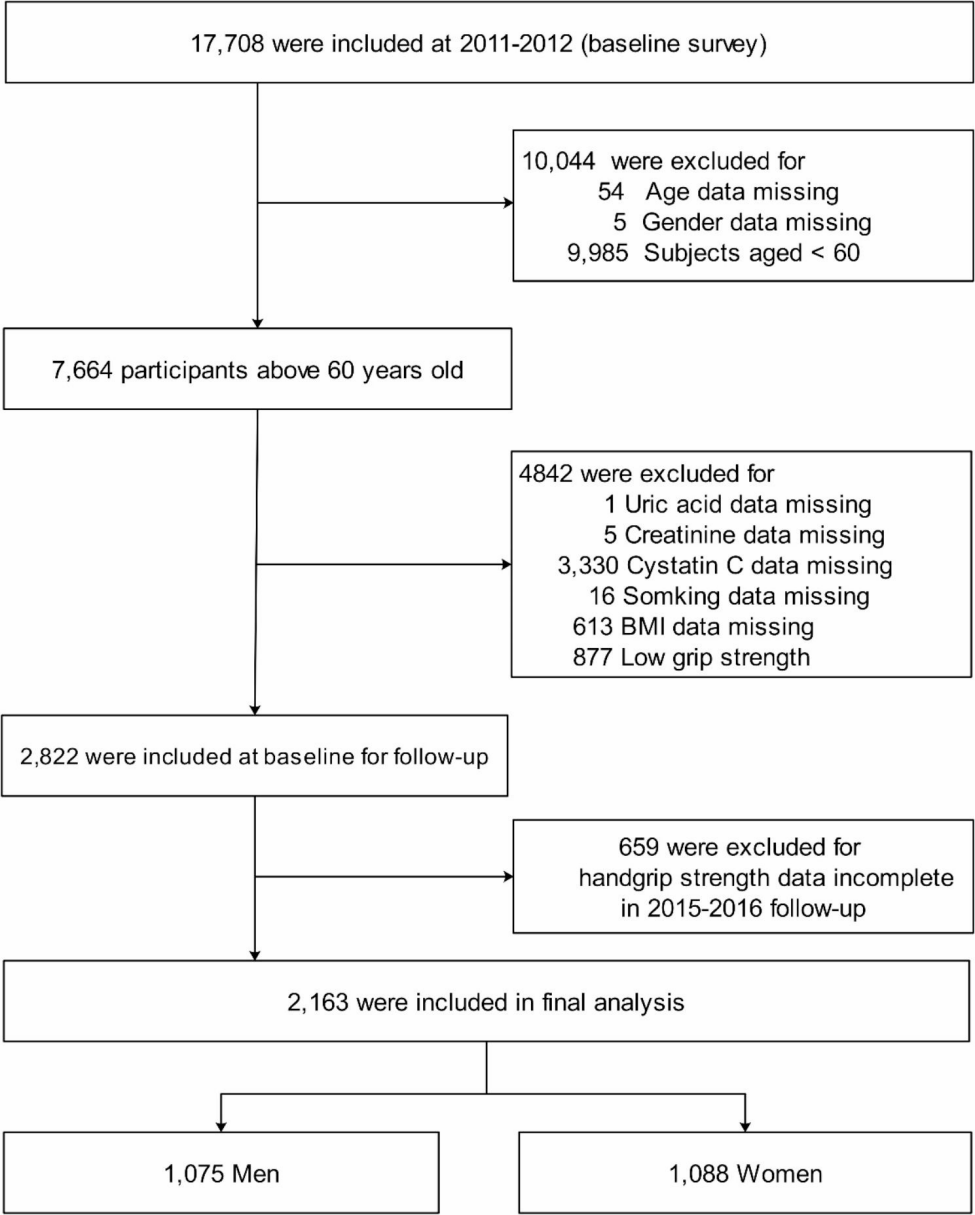
Flow chart of the study participants

The CHARLS survey recruited from 150 counties or districts and 450 villages in 28 provinces in China, generally representing China’s older people annual population. In short, the CHARLS is a nationally representative longitudinal survey focusing on individuals over age 45 residing in diverse communities across China. Initially, our study included 17,708 participants aged over 45 who were enrolled in 2011 at the baseline. However, after excluding 14,886 participants, we were left with 2,822 individuals eligible for follow-up. Participants were excluded based on certain criteria, including incomplete demographic data (N = 59), age under 60 (N = 9,985), incomplete biochemical parameters (N = 3,336), missing lifestyle survey data (N = 16), and absence of anthropometric parameters (N = 613), and low grip strength (N = 877). Additionally, in 2015, 659 participants were excluded due to missing handgrip strength data. As a result, the final analysis comprised 2,163 participants, as shown in Fig. 1. All participants underwent an assessment using a standardized questionnaire interviewed by well-trained staff to collect data on demographic, lifestyle, and health-related information. Detailed information on CHARLS has been published previously [18]. All participants provided informed consent; the Ethical Review Committee of Peking University approved the study protocol (IRB00001052–11,015).

### Measurement of SUA and eGFR

Venous blood samples were collected from each participant after 8 h of fasting in wave 2011. SUA levels (mg/dL) were analyzed using enzymatic-colorimetric methods. The detection limits were up to 20 mg/dL, and the coefficient of variation (CV) intra-assay and inter-assay was equal to 1.10% and 1.90%, respectively. The estimated glomerular filtration rate (eGFR) was calculated based on the result of a multicenter study in Chinese populations [19]. The equation is eGFR = 173.9×CysC^− 0.725^×Cr^− 0.184^×Age^− 0.193^ × 0.89 [if female]. An eGFR ≥ 60 (mL/min/1.73 m^2^) is defined as a high eGFR level, and vice versa.

### Assessment of muscle strength

Muscle strength was evaluated through handgrip strength, measured in kilograms (kg) using a dynamometer (YuejianTM WL-1000, Nantong, China). Participants performed the test while sitting or standing, holding the dynamometer with one hand at a 90° elbow flexion, and exerted maximum force for a brief period. The best measurement from either the right or left hand was recorded as the handgrip strength. According to a recent study concerning low muscle strength in older Chinese adults, the established cutoff points were 28.5 kg for men and 18.6 kg for women [20].

### Study variables

The present study variables included demographic information, anthropometric parameters, and blood sample. Demographic information was collected by trained staff during face-to-face interviews, including age, gender, education, smoking habits, alcohol consumption, and medical history of self-reported diagnosis. Smoking habits indicated whether the respondent reports ever smoking. Drinking consumption was defined whether the participants have had any alcoholic beverage in the past 12 months. Medical history encompasses the respondents’ self-reported answers to whether a doctor has previously diagnosed them with a specific medical condition, including hypertension, diabetes, dyslipidemia, cancer, liver disease, and kidney disease. Other biomarkers, including anthropometric parameters and blood, were collected by China CDC staff. Body mass index (BMI) was calculated as weight(kg) divided by the square of height (m^2^). Waist circumferences (cm) were measured using soft tape around the navel. As mentioned above, blood samples were collected after 8 h of fasting. These biochemical parameters included high-sensitivity CRP (hs-CRP), glycosylated hemoglobin (HbA1c), total cholesterol (TC), triglycerides (TG), HDL cholesterol (HDL-c), LDL cholesterol (LDL-c), creatinine (Cr), and cystatin C (Cys C).

### Statistical analysis

The continuous variables were expressed as mean ± standard deviations (SDs) for normal distribution, and the categorical variables were addressed as frequency and proportion. Those covariates with skewed distribution were presented as median (P25, P75), including hs-CRP and TG. For categorical variables, the potential differences among groups were employed by one-way analysis of variance (ANOVA)(normal distribution) or KruskalWallis rank sum test (skewed distribution). For the categorical variables, we employed the chi-squared test to identify any significant differences across various groups. The association between serum uric acid (UA) and low muscle strength (LMS) was assessed by calculating odds ratios (ORs) and corresponding 95% confidence intervals (CIs) using multivariate logistic regression models. A generalized additive model was employed for interaction analysis and calculated the odds ratio (OR) and 95% confidence interval (CI) for the relationship between SUA per-SD (mg/dL) increased and LMS by different eGFR. We progressively adjusted for age, education levels, smoking habits, drinking consumption, BMI, waist circumferences, medical history (hypertension, diabetes, dyslipidemia, cancer, liver disease, and kidney disease), lipid profiles (TC, TG, HDL-c, and LDL-c), hemoglobin, HbA1c, and hs-CRP. After adjusting for the abovementioned factors, the smoothing plots were illustrated to explore the possible non-linear association between SUA and LMS stratified by eGFR levels.

We conducted a sensitivity analysis using the VIF (variance inflation factor) method to assess the potential impact of multicollinearity between glycated hemoglobin and diabetes, as well as between serum lipids and dyslipidemia.

𝑃 < 0.05 was considered statistically significant. All the statistical analyses were performed using EmpowerStats (http://empowerstats.com/en/; X&Y Solutions, Inc., Boston, MA, USA) and the R package (4.2.0 version).

## Results

The participants were divided into groups based on the quartiles of UA levels separately for males and females, as shown in Table 1. Overall, the average blood uric acid levels are higher in males compared to females (5.02 (mg/dL) ± 1.29 in male vs. 4.16 (mg/dL) ± 1.12 in female, *P* < 0.001). Women have higher eGFR levels than men (74.91 (mL/min/1.73 m^2^) ± 13.70 in male vs. 83.57 (mL/min/1.73 m^2^) ± 16.33 in female, *P* < 0.001). Both genders experience a decrease in estimated glomerular filtration rate (eGFR) as blood uric acid levels increase. Females tend to have a higher proportion of non-smokers and non-drinkers in comparison to males. Moreover, in females, there is an observed increase in grip strength as blood uric acid levels rise.

**Table 1:** Baseline characteristics of participants according to the quartiles of uric acid and gender

Characteristics	Uric acid (mg/dL) in Male (n = 1075) ^*^				*P*-value	Uric acid (mg/dL) in Female(n = 1088) ^**^				*P*-value
	Q1	Q2	Q3	Q4		Q1	Q2	Q3	Q4	
Number	268	269	268	270		271	272	273	272	
Age (years)	66.97 ± 5.30	67.71 ± 5.72	67.43 ± 5.64	67.92 ± 5.54	0.224	66.43 ± 5.90	66.97 ± 5.61	67.73 ± 6.39	67.80 ± 6.03	0.022
Education (%)					0.233					0.775
Illiteracy	58 (21.64%)	45 (16.73%)	44 (16.42%)	36 (13.38%)		146 (53.87%)	149 (54.78%)	143 (52.38%)	142 (52.21%)	
Primary school	138 (51.49%)	152 (56.51%)	155 (57.84%)	165 (61.34%)		102 (37.64%)	103 (37.87%)	99 (36.26%)	107 (39.34%)	
Secondary school above	72 (26.87%)	72 (26.77%)	69 (25.75%)	68 (25.28%)		23 (8.49%)	20 (7.35%)	31 (11.36%)	23 (8.46%)	
Smoking habits (%)					0.117					0.021
No	65 (24.25%)	59 (21.93%)	69 (25.75%)	83 (30.74%)		228 (84.13%)	249 (91.54%)	248 (90.84%)	245 (90.07%)	
Yes	203 (75.75%)	210 (78.07%)	199 (74.25%)	187 (69.26%)		43 (15.87%)	23 (8.46%)	25 (9.16%)	27 (9.93%)	
Drinking history (%)					0.171					0.393
No	139 (51.87%)	146 (54.28%)	125 (46.64%)	125 (46.30%)		243 (89.67%)	240 (88.24%)	240 (87.91%)	231 (84.93%)	
Yes	129 (48.13%)	123 (45.72%)	143 (53.36%)	145 (53.70%)		28 (10.33%)	32 (11.76%)	33 (12.09%)	41 (15.07%)	
Hypertension (%)					< 0.001					< 0.001
Yes	46 (17.29%)	77 (28.62%)	71 (26.49%)	110 (40.74%)		61 (22.59%)	85 (31.60%)	98 (35.90%)	116 (42.65%)	
No	220 (82.71%)	192 (71.38%)	197 (73.51%)	160 (59.26%)		209 (77.41%)	184 (68.40%)	175 (64.10%)	156 (57.35%)	
Dyslipidemia (%)					0.295					0.005
Yes	16 (6.06%)	28 (10.57%)	25 (9.54%)	24 (9.13%)		30 (11.28%)	20 (7.55%)	33 (12.31%)	47 (17.54%)	
No	248 (93.94%)	237 (89.43%)	237 (90.46%)	239 (90.87%)		236 (88.72%)	245 (92.45%)	235 (87.69%)	221 (82.46%)	
Diabetes (%)					0.254					0.059
Yes	13 (4.92%)	15 (5.60%)	8 (3.00%)	18 (6.72%)		22 (8.24%)	22 (8.18%)	10 (3.68%)	25 (9.26%)	
No	251 (95.08%)	253 (94.40%)	259 (97.00%)	250 (93.28%)		245 (91.76%)	247 (91.82%)	262 (96.32%)	245 (90.74%)	
Cancer (%)					0.799					0.956
Yes	2 (0.75%)	3 (1.12%)	1 (0.37%)	2 (0.74%)		2 (0.74%)	2 (0.74%)	3 (1.10%)	2 (0.74%)	
No	264 (99.25%)	264 (98.88%)	266 (99.63%)	268 (99.26%)		268 (99.26%)	267 (99.26%)	269 (98.90%)	268 (99.26%)	
Liver disease (%)					0.220					0.602
Yes	10 (3.76%)	7 (2.62%)	9 (3.36%)	16 (5.97%)		11 (4.09%)	8 (2.96%)	14 (5.20%)	10 (3.68%)	
No	256 (96.24%)	260 (97.38%)	259 (96.64%)	252 (94.03%)		258 (95.91%)	262 (97.04%)	255 (94.80%)	262 (96.32%)	
Kidney disease (%)					0.762					0.488
Yes	23 (8.71%)	19 (7.09%)	18 (6.72%)	23 (8.58%)		11 (4.07%)	12 (4.43%)	18 (6.67%)	16 (5.90%)	
No	241 (91.29%)	249 (92.91%)	250 (93.28%)	245 (91.42%)		259 (95.93%)	259 (95.57%)	252 (93.33%)	255 (94.10%)	
HbA1C (mg/dL)	5.27 ± 0.81	5.22 ± 0.64	5.18 ± 0.61	5.28 ± 0.71	0.295	5.39 ± 0.94	5.31 ± 0.80	5.35 ± 0.92	5.37 ± 0.72	0.708
TC (mg/dL)	180.52 ± 36.75	187.29 ± 35.32	187.01 ± 36.44	193.05 ± 38.07	0.001	200.61 ± 36.97	199.53 ± 36.05	206.26 ± 40.31	208.58 ± 43.62	0.018
TG (mg/dL)	83.19 (60.18-109.52)	88.50 (69.03-126.56)	90.71 (65.49-134.96)	106.20 (77.00-157.53)	< 0.001	103.55 (72.13-137.62)	107.53 (81.20-145.36)	123.90 (87.61-182.31)	134.52 (92.92-193.59)	< 0.001
HDL-c (mg/dL)	52.52 ± 15.85	51.89 ± 17.16	51.70 ± 16.11	50.12 ± 16.23	0.368	55.42 ± 15.96	53.27 ± 14.04	50.30 ± 13.50	49.25 ± 15.61	< 0.001
LDL-c (mg/dL)	109.55 ± 31.85	114.83 ± 30.51	113.86 ± 35.66	116.18 ± 37.31	0.125	123.06 ± 32.97	121.52 ± 33.54	126.63 ± 38.06	125.57 ± 43.01	0.361
Hemoglobin (g/dL)	14.92 ± 2.26	14.89 ± 1.99	14.93 ± 2.49	14.95 ± 2.14	0.992	13.75 ± 2.01	13.71 ± 2.31	13.95 ± 2.27	13.47 ± 1.87	0.080
hs-CRP (mg/dL)	0.84 (0.49–1.90)	1.18 (0.61–2.31)	1.23 (0.63–2.50)	1.44 (0.69–2.84)	< 0.001	0.79 (0.49–1.45)	0.99 (0.61–1.92)	1.28 (0.64–2.52)	1.51 (0.88–3.43)	< 0.001
Uric acid (mg/dL)	3.53 ± 0.48	4.50 ± 0.21	5.29 ± 0.27	6.74 ± 0.87	< 0.001	2.92 ± 0.39	3.69 ± 0.15	4.34 ± 0.23	5.68 ± 0.79	< 0.001
eGFR (mL/min/1.73 m^2^)	81.29 ± 12.30	75.95 ± 12.86	73.52 ± 12.32	68.91 ± 14.27	< 0.001	90.94 ± 14.04	85.34 ± 14.63	80.79 ± 12.57	77.11 ± 18.73	< 0.001
BMI (kg/m^2^)	22.20 ± 4.39	22.43 ± 3.34	22.63 ± 3.58	23.88 ± 3.94	< 0.001	22.44 ± 3.49	22.74 ± 3.53	23.97 ± 3.74	24.26 ± 3.81	< 0.001
WC (cm)	81.73 ± 12.16	83.81 ± 12.48	84.30 ± 10.44	87.04 ± 11.63	< 0.001	82.32 ± 12.10	83.92 ± 12.91	86.37 ± 13.29	88.56 ± 13.42	< 0.001
Grip strength	37.82 ± 6.22	37.56 ± 6.22	38.41 ± 6.52	38.21 ± 6.00	0.388	25.59 ± 4.82	25.93 ± 5.04	26.17 ± 4.91	26.84 ± 5.04	0.027

Note: For categorical variables, the potential differences among groups were employed by one-way analysis of variance (ANOVA) (normal distribution) or KruskalWallis rank sum test (skewed distribution). For the categorical variables, we employed the chi-squared test to identify any significant differences across various groups. eGFR, estimated glomerular filtration rate; BMI, body mass index; WC, waist circumferences; HbA1c, glycosylated hemoglobin; TC, total cholesterol; TG, triglycerides; HDL-c, high-density lipoprotein cholesterol; LDL-c, low-density lipoprotein cholesterol; hs-CRP, high-sensitivity CRP. *The quartile of UA (mg/dL) in male: Q1 < 4.11; 4.11 ≤ Q2 < 4.86; 4.86 ≤ Q3 < 5.78; 5.78 ≤ Q4. **The quartile of UA (mg/dL) in female: Q1 < 3.40; 3.40 ≤ Q2 < 3.97; 3.97 ≤ Q3 < 4.77; 4.77 ≤ Q4. *P* < 0.05 was considered statistically significant

Table 2 presented the association between UA quartiles and low grip strength by genders. In comparison to males, the protective effect of high uric acid levels against low grip strength was only observed in older females, although there may not be a statistically significant difference possibly due to the sample size (OR = 0.69, 95%CI = 0.45–1.04, P = 0.075).

**Table 2:** The association between UA quartiles and Low grip strength in non-adjusted model and full-adjusted model by genders after excluding low grip strength at baseline

	Non-adjusted model		Full-adjusted model	
	OR (95%CI)	P-value	OR (95%CI)	P-value
Male*				
Q1	ref		ref	
Q2	1.19(0.79,1.79)	0.410	1.18(0.76,1.83)	0.463
Q3	1.24(0.83,1.88)	0.296	1.16(0.74,1.82)	0.516
Q4	1.23(0.81,1.85)	0.330	1.19(0.75,1.89)	0.457
Female**				
Q1	ref		Ref	
Q2	0.79(0.55,1.14)	0.215	0.86(0.59,1.27)	0.452
Q3	0.65(0.45,0.95)	0.025	0.67(0.44,1.00)	0.050
Q4	0.65(0.45,0.94)	0.022	0.69(0.45,1.04)	0.075

Full-adjusted model including age, education levels, smoking, drinking, BMI, WC, medical history, HbA1c, lipid profiles, hemoglobin, and hs-CRP. The odds ratios (OR) represent the odds of low muscle strength with the first quartile of UA as the baseline category. Medical history including hypertension, diabetes, dyslipidemia, cancer, liver disease, and kidney disease; Lipid profiles including total cholesterol, triglycerides, high-density lipoprotein cholesterol, and low-density lipoprotein cholesterol; BMI, body mass index; WC, waist circumferences; HbA1c, glycosylated hemoglobin; hs-CRP, high-sensitivity CRP; SUA, serum uric acid; eGFR, estimated glomerular filtration rate; SD, standard deviation; OR, odds ratio; CI, confidence interval. *The quartile of UA (mg/dL) in male: Q1 < 4.11; 4.11 ≤ Q2 < 4.86; 4.86 ≤ Q3 < 5.78; 5.78 ≤ Q4. **The quartile of UA (mg/dL) in female: Q1 < 3.40; 3.40 ≤ Q2 < 3.97; 3.97 ≤ Q3 < 4.77; 4.77 ≤ Q4

Table 3 presented the stratification of eGFR into high and low groups to explore its interaction with SUA concerning low grip strength. In females, the association between a high eGFR level and serum uric acid remained significant in the low grip strength, after progressively adjusting for several risk factors (OR = 0.82, 95%CI = 0.70–0.97, P = 0.021). In contrast, such a relationship was not observed in men (OR = 1.08, 95%CI = 0.94–1.25, P = 0.2714). Additionally, we detected a statistically significant interaction between different eGFR levels in women (P = 0.034) in contrast to the interaction analysis in men (P = 0.445).

**Table 3:** Association of SUA perSD increases with the risk of low muscle strength in different eGFR levels by genders

	Male					Female				
	Low eGFR		High eGFR			Low eGFR		High eGFR		
model	OR (95%CI)	*P*-value*	OR (95%CI)	*P*-value*	P for interaction*	OR (95%CI)	*P*-value*	OR (95%CI)	*P*-value*	P for interaction*
Not adjusted	0.89(0.66, 1.20)	0.437	1.05(0.93, 1.19)	0.433	0.304	1.09(0.85, 1.41)	0.486	0.79(0.68, 0.91)	0.002	0.028
Plus age and education	0.96(0.70, 1.32)	0.804	1.05(0.92, 1.20)	0.469	0.469	1.12(0.86, 1.45)	0.405	0.77(0.66, 0.90)	0.001	0.017
Plus smoking and drinking	0.97(0.71, 1.33)	0.865	1.06(0.92, 1.21)	0.427	0.637	1.12(0.86, 1.45)	0.404	0.78(0.66, 0.90)	0.001	0.018
Plus BMI and waist	0.99(0.72, 1.35)	0.926	1.07(0.93, 1.23)	0.332	0.635	1.11(0.85, 1.45)	0.443	0.81(0.69, 0.94)	0.007	0.042
Plus medical history	0.98(0.72, 1.35)	0.916	1.06(0.92, 1.22)	0.398	0.661	1.10(0.84, 1.43)	0.491	0.79(0.68, 0.93)	0.005	0.040
Plus HbA1c	0.95(0.69, 1.30)	0.734	1.06(0.92, 1.22)	0.412	0.519	1.10(0.84, 1.43)	0.498	0.79(0.67, 0.93)	0.005	0.040
Plus lipid profiles	0.95(0.69, 1.32)	0.777	1.08(0.94, 1.25)	0.266	0.473	1.15(0.88, 1.51)	0.306	0.82(0.69, 0.96)	0.017	0.032
Plus Hemoglobin	0.93(0.67, 1.30)	0.687	1.09(0.95, 1.26)	0.212	0.378	1.16(0.88, 1.52)	0.295	0.83(0.70, 0.97)	0.023	0.035
Plus hs-CRP	0.94(0.68, 1.31)	0.732	1.08(0.94, 1.25)	0.271	0.445	1.16(0.88, 1.52)	0.293	0.82(0.70, 0.97)	0.021	0.034

The generalized additive model progressively adjusted risk factors and smoothly adjusted for non-linear factors in the last model. High eGFR, eGFR ≥ 60 mL/min/1.73 m^2^. Medical history including hypertension, diabetes, dyslipidemia, cancer, liver disease, and kidney disease; Lipid profiles including total cholesterol, log triglycerides, high-density lipoprotein cholesterol, and low-density lipoprotein cholesterol; BMI, body mass index; WC, waist circumferences; HbA1c, glycosylated hemoglobin; hs-CRP, log high-sensitivity CRP; SUA, serum uric acid; SD, standard deviation; OR, odds ratios; CI, confidence interval

*P < 0.05 was considered statistically significant

**Fig. 2:**
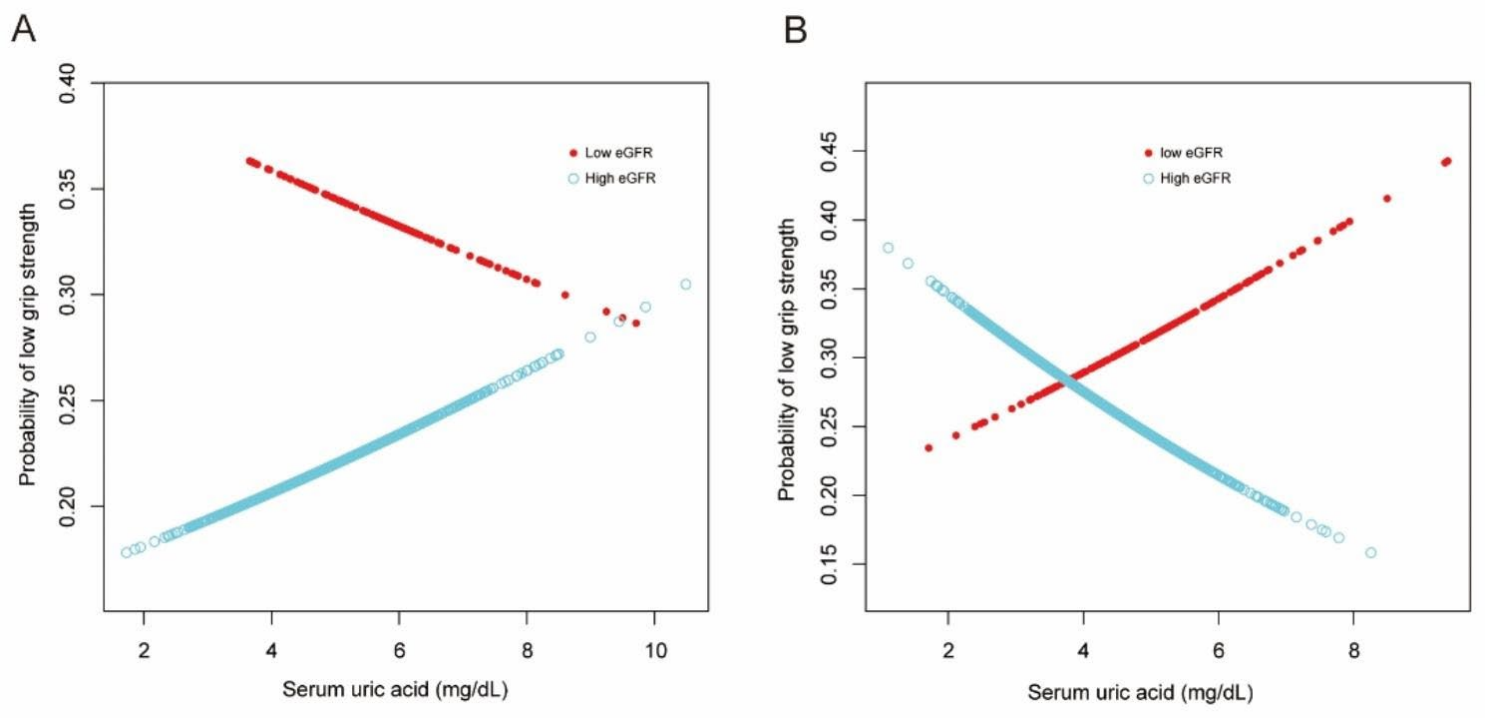
The association between serum uric acid and the risk of low muscle strength stratification by different eGFR levels in the smoothing plot with full adjustment in male (**A**) and female (**B**)

Figure 2 demonstrated the association between SUA levels and the risk of LMS through a smoothing plot. This analysis incorporated adjustments for the aforementioned risk factors and involves stratification based on different eGFR levels. The findings unveiled a significant trend among female subjects, wherein an elevated uric acid concentration exhibited a protective effect against the occurrence of low muscle strength, but only in conjunction with a higher estimated glomerular filtration rate. However, this trend was not observed in male participants.

The sensitivity analysis indicated that there was multicollinearity in TC and LDL, as shown in Table S1. However, compared to the original model, removing TC did not affect the association between serum uric acid and grip strength, regardless of gender, as shown in Table S2.

## Discussion

To the best of our knowledge, this was the first cohort study that investigated the causal relationship between serum uric acid and the risk of low muscle strength at different eGFR levels above 60 years population. Our results suggested that high uric acid levels only provide a protective effect against low muscle strength when accompanied by high eGFR levels, especially in older females.

These results help to clarify conflicts among previous studies. Some cross-sectional studies reported a positive association between higher uric acid levels and better grip strength [14, 21]. However, these studies did not account for the influence of eGFR or adjust for eGFR as a confounding factor, potentially leading to misleading conclusions regarding the effect of uric acid on muscle strength. In contrast, our study at baseline excluded individuals who already had low muscle strength to minimize the impact of this population, ensuring a causal relationship between uric acid and muscle strength. Our study is in line with the findings of a Korean study that excluded populations with eGFR < 60 and demonstrated a positive association between SUA and grip strength in older people [22]. Moreover, Nahas et al. also adjusted for eGFR and other confounders, showing that older men and women may benefit from higher SUA levels for better handgrip strength [15]. Thus, our study, along with the abovementioned studies, suggests that a high eGFR level may act as a protective factor, allowing serum uric acid levels to positively impact muscular strength.

We have observed that different eGFR levels can lead to contrasting effects of SUA on the risk of LMS in both men and women. However, the underlying mechanism by which a high serum level of SUA is associated with a decreased risk of LMS in varying eGFR conditions remains uncertain. Many studies have proposed that SUA may exhibit antioxidant properties by scavenging reactive oxygen species (ROS), thereby reducing oxidative stress [12–16, 21, 22]. Nevertheless, this hypothesis may require further exploration, especially concerning lower eGFR levels. The accumulation of SUA due to declining kidney function may be influenced by alterations in the body’s chemical environment, potentially affecting the antioxidant ability of uric acid [23]. For instance, the accumulation of bicarbonate, often seen in renal insufficiency-related electrolyte disturbances, might compromise uric acid’s capacity to counteract tyrosine nitrosylation, a crucial mechanism of oxidative damage [24].

Furthermore, recent research suggested that uric acid may contribute to oxidative stress rather than acting as an antioxidant under physiological conditions [11]. It has been established that uric acid can directly contribute to the production of ROS, and the antioxidant properties of uric acid may be offset by ROS generated from xanthine oxidoreductase-catalyzed reactions. This suggests that only the administration of exogenous uric acid might exhibit an antioxidative stress effect. For example, studies have demonstrated improved clinical outcomes in patients with acute ischemic stroke following uric acid administration [25], and in mice with Parkinson’s disease, uric acid injections have shown a neuroprotective effect [26]. Consequently, the accumulation of oxidative stress may play a role in the reduction of age-related declines in muscle strength [27] and kidney function [8]. However, since accurate detection of circulating xanthine oxidoreductase activity is not yet prevalent in clinical settings and there are limited studies exploring exogenous uric acid’s impact on muscle strength improvement, further investigation is required to determine whether uric acid acts as an antioxidant in muscle wasting associated with aging.

Our findings also revealed that the impact of SUA on LMS in different eGFR levels is sex specific. Specifically, women with high eGFR levels and higher serum uric acid showed a 18% lower risk of LMS, which contrasts with the results of a previous study. Veronese et al. conducted a cohort study and adjusted for eGFR and other confounders, reporting that hyperuricemia in men was associated with lower handgrip strength, while this relationship was not observed in women [16]. Gender differences play a critical role in the associations between physical activity and muscle strength during aging [28]. The consensus of Asian working group for sarcopenia 2019 revealed that sarcopenia was more prevalent in men than women, suggesting that sexual dimorphism may influence the disease’s pathogenesis [2].

Sex hormones may contribute to the differences between genders, affecting circulating uric acid levels, eGFR, and muscle strength. During adolescence, higher testosterone and lower sex hormone-binding globulin have been reported as gender differences in circulating uric acid [29]. With aging, testosterone levels decrease, impacting body composition, including reduced muscle mass, decreased strength, and increased muscular fatigue [30]. High follicle-stimulating hormone has been linked to declined eGFR in post-menopausal women [31]. A mendelian randomization study from the United Kingdom Biobank population suggested that high sex hormone-binding globulin was associated with better kidney function and a lower risk of chronic kidney disease (CKD) in men [32]. A prospective cohort study by Tsai et al., using data from the MJ Health Screening Database, indicated that a low serum testosterone level (< 400 ng/dL) was significantly associated with a high SUA level (> 7 mg/dL) in males [33]. Furthermore, testosterone therapy could pharmacologically increase serum uric acid [34], possibly through elevated UA production by simulating xanthine oxidase [35]. Testosterone administration has also been shown to increase muscle mass [36], muscle strength, and muscle power [37]. These studies collectively suggest that testosterone may contribute to a gender-specific difference in the relationship between SUA and LMS. Therefore, the interaction of sex differences in muscular strength and circulating uric acid in kidney function warrants further attention and investigation.

Despite the theory of sex hormones and oxidative stress, other possibilities could be affecting muscular strength with SUA level. First, sarcopenia has been linked to chronic low-grade inflammation [38], and high SUA levels resulting from decreased renal excretion might lead to the formation of urate crystals [39], potentially inducing conditions like gout and nephrolithiasis. These conditions can lead to an increase in circulating inflammatory mediators. A meta-analysis has shown that higher circulating inflammatory markers, such as CRP and IL-6, were associated with a decline in muscular strength [40]. However, in the present study, after adjusting for hs-CRP, the results remained consistent, suggesting that inflammation may not be the primary driver of muscle strength loss in this context. This finding is in line with a Danish cohort study that also showed a weak relationship between high hs-CRP levels and low muscular mass [41]. Second, metabolic syndrome has been associated with lower muscular strength [27], and high waist circumference has been positively correlated with reduced handgrip strength [42]. Nevertheless, in our study, the results remained robust even after adjusting for various metabolic factors, including BMI, waist circumference, lipid profiles, HbA1c, and medical history separately. This indicates that metabolic syndrome may not be the crucial factor influencing the relationship between SUA and LMS in different eGFR levels. Third, decreased hemoglobin levels have been associated with sarcopenia in non-dialysis chronic kidney disease patients [43] and kidney transplant recipients [44]. In the present study, adjusting for hemoglobin levels did not alter the results in women and men.

### Limitations

Although our hypotheses were supported statistically, our study’s results should be interpreted within its limitations. First, we did not evaluate whether the SUA levels changed during the follow-up and investigated the potential confounders of SUA levels at the baseline, including allopurinol and diuretics. Second, despite adjusting significant covariates separately, we cannot exclude the residual confounding of unmeasured factors in an observational study, including sex hormones, biomarkers of oxidative stress, nutritional status, and daily activity. Third, we did not have any information about gout history in this cohort. Moreover, the results should not be extrapolated to the presence of gout, which was related to SUA level and may affect the performance of muscle strength testing. Fourth, we focused on the population above 60yrs, which means that the results should not be extrapolated to those under 60 years, as they were “healthier” than older people.

## Conclusion

The population-based cohort study conducted in Chinese older adults has shed light on the association between high SUA levels and muscle strength. This study emphasized the importance of considering sex-specific differences in these associations and suggest that high SUA levels, in conjunction with high eGFR, may have a protective effect on muscle strength in older women. Further research is warranted to explore the underlying mechanisms that drive these associations and to confirm the potential benefits of maintaining appropriate SUA levels in older people, especially females, to support muscle health.

### Electronic supplementary material

Below is the link to the electronic supplementary material.

Supplementary Material 1

## Data Availability

The datasets generated and analyzed during the current study are available in the CHARLS database, which is publicly available on the CHARLS website (http://charls.pku.edu.cn/).

## Abbreviations

SUA: Serum uric acid
eGFR: estimated glomerular filtration rate
LMS: Low muscle strength
BMI: Body mass index
CHARLS: China Health and Retirement Longitudinal Study ORs:odds ratio
CI: confidence interval
CV: coefficient of variation
hs-CRP: high-sensitivity CRP
HbA1c: glycosylated hemoglobin
TC: total cholesterol
TG: triglycerides
HDL-c: HDL cholesterol
LDL-c: LDL cholesterol
Cr: creatinine
Cys C: cystatin C

## References

[1] Roubenoff R, Castaneda C (2001). Sarcopenia-understanding the dynamics of aging muscle. JAMA.

[2] Chen LK, Woo J, Assantachai P (2020). Asian Working Group for Sarcopenia: 2019 Consensus Update on Sarcopenia diagnosis and treatment. J Am Med Dir Assoc.

[3] Wang X, Ma Y, Wang J (2016). Mobility and muscle strength together are more strongly correlated with Falls in Suburb-Dwelling Older Chinese. Sci Rep.

[4] Uusi-Rasi K, Karinkanta S, Tokola K, Kannus P, Sievänen H. Bone Mass and Strength and Fall-Related Fractures in Older Age. *J Osteoporos*. 2019; 2019:5134690. 10.1155/2019/5134690. 10.1155/2019/5134690 PMC6754934 31583071

[5] Parra-Soto S, Pell JP, Celis-Morales C, Ho FK (2022). Absolute and relative grip strength as predictors of cancer: prospective cohort study of 445 552 participants in UK Biobank. J Cachexia Sarcopenia Muscle.

[6] Cho SK, Chang Y, Kim I, Ryu S (2018). U-Shaped Association between serum uric acid level and risk of mortality: a Cohort Study. Arthritis Rheumatol.

[7] Wu M, Wei Y, Lv J, et al. Associations of muscle mass, strength, and quality with all-cause mortality in China: a population-based cohort study. Chin Med J [Engl]. 2022. 10.1097. 10.1097/CM9.0000000000002193 PMC9433076 35838536

[8] Hajam YA, Rani R, Ganie SY (2022). Oxidative stress in Human Pathology and Aging: Molecular Mechanisms and Perspectives. Cells.

[9] Howard C, Ferrucci L, Sun K (2007). Oxidative protein damage is associated with poor grip strength among older women living in the community. J Appl Physiol [1985].

[10] Liu N, Xu H, Sun Q et al. The Role of Oxidative Stress in Hyperuricemia and Xanthine Oxidoreductase [XOR] Inhibitors. *Oxid Med Cell Longev*. 2021; 2021:1470380. 10.1155/2021/1470380. 10.1155/2021/1470380 PMC8019370 33854690

[11] Kurajoh M, Fukumoto S, Yoshida S (2021). Uric acid shown to contribute to increased oxidative stress level independent of xanthine oxidoreductase activity in MedCity21 health examination registry. Sci Rep.

[12] Xu L, Jing Y, Zhao C (2020). Cross-sectional analysis of the association between serum uric acid levels and handgrip strength among chinese adults over 45 years of age. Ann Transl Med.

[13] Huang C, Niu K, Kobayashi Y (2013). An inverted J-shaped association of serum uric acid with muscle strength among japanese adult men: a cross-sectional study. BMC Musculoskelet Disord.

[14] Liu X, Chen X, Hu F (2022). Higher uric acid serum levels are associated with sarcopenia in west China: a cross-sectional study. BMC Geriatr.

[15] Nahas PC, Rossato LT, de Branco FMS, Azeredo CM, Rinaldi AEM, de Oliveira EP (2021). Serum uric acid is positively associated with muscle strength in older men and women: findings from NHANES 1999–2002. Clin Nutr.

[16] Veronese N, Stubbs B, Trevisan C (2017). Results of an Observational Cohort Study of Hyperuricemia as a predictor of poor physical performance in the Elderly. Arthritis Care Res [Hoboken].

[17] An JN, Kim JK, Lee HS, Kim SG, Kim HJ, Song YR (2021). Late stage 3 chronic kidney disease is an independent risk factor for sarcopenia, but not proteinuria. Sci Rep.

[18] Zhao Y, Hu Y, Smith JP, Strauss J, Yang G (2014). Cohort profile: the China Health and Retirement Longitudinal Study [CHARLS]. Int J Epidemiol.

[19] Feng JF, Qiu L, Zhang L (2013). Multicenter study of creatinine- and/or cystatin C-based equations for estimation of glomerular filtration rates in chinese patients with chronic kidney disease. PLoS ONE.

[20] Ge S, Du Q, Feng X (2022). Optimal cutoffs for the diagnosis of Sarcopenia in older chinese adults. Front Nutr.

[21] Molino-Lova R, Sofi F, Pasquini G (2017). Higher uric acid serum levels are associated with better muscle function in the oldest old: results from the Mugello Study. Eur J Intern Med.

[22] Lee J, Hong YS, Park SH, Kang KY. High serum uric acid level is associated with greater handgrip strength in the aged population. Arthritis Res Ther. 2019;211. 10.1186/s13075-019-1858-2. 10.1186/s13075-019-1858-2 PMC6417193 30867037

[23] Sautin YY, Johnson RJ (2008). Uric acid: the oxidant-antioxidant paradox. Nucleosides Nucleotides Nucleic Acids.

[24] Whiteman M, Ketsawatsakul U, Halliwell B (2002). A reassessment of the peroxynitrite scavenging activity of uric acid. Ann N Y Acad Sci.

[25] Chamorro A, Amaro S, Castellanos M (2014). Safety and efficacy of uric acid in patients with acute stroke [URICO-ICTUS]: a randomised, double-blind phase 2b/3 trial. Lancet Neurol.

[26] Huang TT, Hao DL, Wu BN, Mao LL, Zhang J (2017). Uric acid demonstrates neuroprotective effect on Parkinson’s disease mice through Nrf2-ARE signaling pathway. Biochem Biophys Res Commun.

[27] Gonzalez A, Simon F, Achiardi O, Vilos C, Cabrera D, Cabello-Verrugio C (2021). The critical role of oxidative stress in sarcopenic obesity. Oxid Med Cell Longev.

[28] Gómez-Cabello A, Carnicero JA, Alonso-Bouzón C (2014). Age and gender, two key factors in the associations between physical activity and strength during the ageing process. Maturitas.

[29] Wang Y, Charchar FJ (2021). Establishment of sex difference in circulating uric acid is associated with higher testosterone and lower sex hormone-binding globulin in adolescent boys. Sci Rep.

[30] Barone B, Napolitano L, Abate M (2022). The role of Testosterone in the Elderly: what do we know?. Int J Mol Sci.

[31] Li Q, Zheng D, Lin H (2021). High circulating follicle-stimulating hormone level is a potential risk factor for renal dysfunction in Post-Menopausal Women. Front Endocrinol [Lausanne].

[32] Zhao JV, Schooling CM (2021). Sex-specific Associations of sex hormone binding globulin with CKD and kidney function: a univariable and multivariable mendelian randomization study in the UK Biobank. J Am Soc Nephrol.

[33] Tsai MK, Hung KC, Liao CC, Pan LF, Hung CL, Yang DH (2022). The Association between serum testosterone and hyperuricemia in males. J Clin Med.

[34] Kurahashi H, Watanabe M, Sugimoto M (2013). Testosterone replacement elevates the serum uric acid levels in patients with female to male gender identity disorder. Endocr J.

[35] Olatunji LA, Areola ED, Badmus OO (2018). Endoglin inhibition by sodium acetate and flutamide ameliorates cardiac defective G6PD-dependent antioxidant defense in gestational testosterone-exposed rats. Biomed Pharmacother.

[36] Sinclair M, Grossmann M, Hoermann R, Angus PW, Gow PJ (2016). Testosterone therapy increases muscle mass in men with cirrhosis and low testosterone: a randomised controlled trial. J Hepatol.

[37] Storer TW, Woodhouse L, Magliano L (2008). Changes in muscle mass, muscle strength, and power but not physical function are related to testosterone dose in healthy older men. J Am Geriatr Soc.

[38] Schaap LA, Pluijm SM, Deeg DJ (2009). Higher inflammatory marker levels in older persons: associations with 5-year change in muscle mass and muscle strength. J Gerontol A Biol Sci Med Sci.

[39] Li X, Meng X, Timofeeva M et al. Serum uric acid levels and multiple health outcomes: umbrella review of evidence from observational studies, randomised controlled trials, and Mendelian randomisation studies *BMJ*. 2017; 357:j2376. 10.1136/BMJ.j2376. 10.1136/bmj.j2376 PMC5461476 28592419

[40] Tuttle CSL, Thang LAN, Maier AB (2020). Markers of inflammation and their association with muscle strength and mass: a systematic review and meta-analysis. Ageing Res Rev.

[41] Kamper RS, Alcazar J, Andersen LL (2021). Associations between inflammatory markers, body composition, and physical function: the Copenhagen Sarcopenia Study. J Cachexia Sarcopenia Muscle.

[42] Fraser BJ, Blizzard L, Buscot MJ (2022). Muscular strength measured across the life-course and the metabolic syndrome. Nutr Metab Cardiovasc Dis.

[43] de Amorim GJ, Calado CKM, Souza de Oliveira BC, et al. Sarcopenia in Non-Dialysis chronic kidney Disease Patients: Prevalence and Associated factors. Front Med [Lausanne]. 2022;9. 10.3389/fmed.2022.854410. Published 2022 Apr 7. 10.3389/fmed.2022.854410 PMC9021613 35463026

[44] Vinke JSJ, Wouters HJCM, Stam SP (2022). Decreased haemoglobin levels are associated with lower muscle mass and strength in kidney transplant recipients [published online ahead of print, 2022 Jun 3]. J Cachexia Sarcopenia Muscle.

