# Supplementary material for: Association between elevated serum uric acid levels and high estimated glomerular filtration rate with reduced risk of low muscle strength in older people: a retrospective cohort study
Source: BMC Geriatr. 2023 Oct 11;23:652. doi: 10.1186/s12877-023-04374-3 (PMC10568872; doi:10.1186/s12877-023-04374-3)
Supplement: Supplementary file 1 — Supplementary Material 1 [file 12877_2023_4374_MOESM1_ESM.docx]

| Table S1 VIF change in full-adjusted model with and without total cholesterol | | | | |
| --- | --- | --- | --- | --- |
| variables | VIF in full-adjusted model | | VIF in full-adjusted model without TC | |
|  | Male | Female | Male | Female |
| Age | 1.10 | 1.09 | 1.10 | 1.09 |
| Education | 1.07 | 1.07 | 1.07 | 1.07 |
| Smoking | 1.09 | 1.04 | 1.09 | 1.04 |
| Drinking | 1.12 | 1.04 | 1.12 | 1.04 |
| BMI | 1.81 | 1.04 | 1.81 | 1.04 |
| WC | 1.71 | 1.20 | 1.71 | 1.20 |
| hypertension | 1.24 | 1.14 | 1.24 | 1.14 |
| diabetes | 1.35 | 1.25 | 1.35 | 1.25 |
| dyslipidemia | 1.15 | 1.20 | 1.15 | 1.19 |
| cancer | 1.02 | 1.04 | 1.02 | 1.03 |
| liver disease | 1.03 | 1.01 | 1.03 | 1.01 |
| kidney disease | 1.04 | 1.05 | 1.03 | 1.05 |
| HbA1c | 1.34 | 1.21 | 1.34 | 1.20 |
| TC | 18.08 | 12.89 | - | - |
| TG | 4.55 | 6.03 | 1.33 | 1.52 |
| HDL | 4.49 | 3.17 | 1.37 | 1.49 |
| LDL | 14.58 | 11.00 | 1.05 | 1.11 |
| hemoglobin | 1.13 | 1.08 | 1.11 | 1.08 |
| hs-CRP | 1.02 | 1.04 | 1.02 | 1.03 |
| Mean VIF | 3.05 | 2.54 | 1.21 | 1.14 |
| VIF, variance inflation factor; WC, waist circumferences; hs-CRP, high-sensitivity CRP; HbA1c, glycosylated hemoglobin; TC, total cholesterol; TG, triglycerides; HDL, high-density lipoprotein; LDL, low-density lipoprotein. | | | | |

| Table S2. The association between UA quartiles and low grip strength in full-adjusted model with or without total cholesterol by genders | | | | |
| --- | --- | --- | --- | --- |
|  | Full-adjusted model with TC | | Full-adjusted model without TC | |
|  | OR (95%CI) | P-value | OR (95%CI) | P-value |
| Male* | | | | |
| Q1 | ref | - | ref | - |
| Q2 | 1.18(0.76,1.83) | 0.463 | 1.17(0.75,1.82) | 0.479 |
| Q3 | 1.16(0.74,1.82) | 0.516 | 1.15(0.73,1.80) | 0.541 |
| Q4 | 1.19(0.75,1.89) | 0.457 | 1.16(0.74,1.84) | 0.517 |
| Female** | | | | |
| Q1 | ref | - | Ref | - |
| Q2 | 0.86(0.59,1.27) | 0.452 | 0.86(0.59,1.27) | 0.457 |
| Q3 | 0.67(0.44,1.00) | 0.050 | 0.67(0.45,1.00) | 0.050 |
| Q4 | 0.69(0.45,1.04) | 0.075 | 0.69(0.45,1.04) | 0.076 |
| Full-adjusted model including age, education levels, smoking, drinking, BMI, WC, medical history, HbA1c, lipid profiles, hemoglobin, and hs-CRP. The odds ratios (OR) represent the odds of low muscle strength with the first quartile of UA as the baseline category. Medical history including hypertension, diabetes, dyslipidemia, cancer, liver disease, and kidney disease; Lipid profiles including total cholesterol (TC), triglycerides (TG), high-density lipoprotein cholesterol, and low-density lipoprotein cholesterol; BMI, body mass index; WC, waist circumferences; HbA1c, glycosylated hemoglobin; hs-CRP, high-sensitivity CRP; SUA, serum uric acid; eGFR, estimated glomerular filtration rate; SD, standard deviation; OR, odds ratio; CI, confidence interval. *The quartile of UA (mg/dL) in male: Q1<4.14; 4.14≤Q2<4.87; 4.87≤Q3<5.79; 5.79≤Q4. **The quartile of UA (mg/dL) in female: Q1<3.36; 3.36≤Q2<3.96; 3.96≤Q3<4.77; 4.77≤Q4. | | | | |
